# Structure of Ty1 Internally Initiated RNA Influences Restriction Factor Expression

**DOI:** 10.3390/v9040074

**Published:** 2017-04-10

**Authors:** Leszek Błaszczyk, Marcin Biesiada, Agniva Saha, David J. Garfinkel, Katarzyna J. Purzycka

**Affiliations:** 1 Institute of Bioorganic Chemistry, Polish Academy of Sciences, Poznan 61-704, Poland; blaszcz@ibch.poznan.pl (L.B.); biesiada@ibch.poznan.pl (M.B.); 2 Department of Biochemistry & Molecular Biology, University of Georgia, Athens, GA 30602, USA; agniva.saha@gmail.com (A.S.); djgarf@uga.edu (D.J.G.)

**Keywords:** RNA structure, Ty1 retrotransposon, Gag, translation regulation

## Abstract

The long-terminal repeat retrotransposon Ty1 is the most abundant mobile genetic element in many *Saccharomyces cerevisiae* isolates. Ty1 retrotransposons contribute to the genetic diversity of host cells, but they can also act as an insertional mutagen and cause genetic instability. Interestingly, retrotransposition occurs at a low level despite a high level of Ty1 RNA, even though *S. cerevisiae* lacks the intrinsic defense mechanisms that other eukaryotes use to prevent transposon movement. p22 is a recently discovered Ty1 protein that inhibits retrotransposition in a dose-dependent manner. p22 is a truncated form of Gag encoded by internally initiated Ty1i RNA that contains two closely-spaced AUG codons. Mutations of either AUG codon compromise p22 translation. We found that both AUG codons were utilized and that translation efficiency depended on the Ty1i RNA structure. Structural features that stimulated p22 translation were context dependent and present only in Ty1i RNA. Destabilization of the 5′ untranslated region (5′ UTR) of Ty1i RNA decreased the p22 level, both in vitro and in vivo. Our data suggest that protein factors such as Gag could contribute to the stability and translational activity of Ty1i RNA through specific interactions with structural motifs in the RNA.

## 1. Introduction

Ty1 is a long-terminal repeat (LTR) retrotransposon in the *Pseudoviridae* family and the most abundant mobile genetic element in the *Saccharomyces cerevisiae* reference strain [1]. Ty1 contains *GAG* and *POL* genes bracketed by LTRs and proliferates in the yeast genome by integrating new copies through an RNA-mediated mechanism [2]. Dimeric Ty1 RNA is present in virus-like particles (VLPs) [3] that are comprised of the capsid protein Gag and Gag-Pol; the latter being synthesized by a programmed +1 frameshift event that occurs at overlapping leucine codons in *GAG* and *POL* [4]. *POL* encodes protease (PR), reverse transcriptase (RT) and integrase (IN), which are required for protein maturation, reverse transcription and integration, respectively. Gag is a VLP structural component and is expressed as a 441-amino acid precursor (p49) that undergoes a C-terminal cleavage by PR to produce the mature 401-residue protein (p45). Ty1 Gag binds RNA in vitro [5,6] and serves as a multifunctional regulator that orchestrates retrotransposon replication [7]. 

Ty1 contributes to the genetic diversity of *S. cerevisiae* and closely related species, however, these elements can also act as insertional mutagens and cause genetic instability by recombination-mediated gene rearrangements. Overloading the genome with retrotransposon insertions is another scenario that could be lethal to the cell. Paradoxically, Ty1 retrotransposition occurs at low rate, despite a high level of Ty1 RNA [2]. *S. cerevisiae* also lack the intrinsic defense mechanisms to prevent retrotransposition that are typically active in other eukaryotes, including DNA methylation [8,9], and the expression of several host proteins, such as apolipoprotein B mRNA-editing enzyme catalytic polypeptide-like 3 (APOBEC3) family members [10] or RNAi components [11,12]. Early on, a region of Ty1 required for copy number control (CNC) was identified but the mechanism underlying CNC remained puzzling [13]. Recent genetic analysis of the CNC region identified mutations abrogating CNC that map within *GAG* downstream of two internal AUG codons [14,15]. The separation of function phenotype displayed by one of the *GAG* mutations suggests that Ty1 encodes a protein that restricts its movement. Indeed, the recently discovered protein p22 inhibits retrotransposition in a dose-dependent manner and mediates CNC. p22 is encoded by the C-terminal half of Ty1 *GAG*, and similar to Gag-p49, undergoes maturation by Ty1 protease to form p18. However, p22 is encoded by internally initiated Ty1i RNA that contains two closely spaced AUG codons. Ribosomal profiling analyses show preferential usage of AUG1, but mutational analysis of Ty1i RNA initiation codons AUG1 and AUG2 suggests that both have the potential to be utilized for p22 translation. p18 expressed from either AUG1 or AUG2 confers strong inhibition of Ty1 mobility that correlates with their level of expression. Also, p22/p18 target Gag and inhibit several steps in the process of retrotransposition prior to reverse transcription [14,15,16]. 

Like programmed Ty1 frameshifting, employing multiple start codons to initiate the synthesis of p22 is reminiscent of the non-canonical translation strategies that viruses use to maximize their coding potential [17]. Canonical 5′-end-dependent translation initiation generally permits only one protein to be synthesized from a particular mRNA. However, the leaky scanning mechanism allows the production of functionally distinct proteins from a single transcript containing multiple initiation codons. In these cases, a suboptimal sequence surrounding the first AUG codon limits its recognition, which allows ribosomal scanning and translation from downstream initiation codons [17]. This strategy is commonly employed by RNA viruses, including retroviruses [18]. 

We have shown that p22 translation is a cap-dependent event, however, our results suggest that the structure of 5′ UTR of Ty1i mRNA may contribute to the efficiency of translation [14]. Secondary and tertiary structures of 5′ UTRs play important roles in the regulation of translation by affecting the recruitment, positioning and movement of ribosomes [19]. Folding of the 5′ UTR into an ensemble of secondary structures may influence the initiation of translation either positively or negatively. The nature of this effect is attributed, at least in part, to the thermodynamic stability of the structural elements formed in the 5′ UTR, their guanine-cytosine (GC) content, and positioning in relation to the 5′ cap and AUG initiation codon. Hairpin structures of even moderate thermodynamic stability located close to the 5′-end of the mRNA prevent cap-dependent formation of the preinitiation complexes and can lead to translation inhibition [20,21,22,23]. On the other hand, secondary structures present in the coding region may stimulate translation if placed at particular distances downstream of the initiation codon [24,25]. This stimulatory effect may be caused by a hairpin structure that pauses migration of the preinitiation complexes. Hairpin structures can be important for mRNAs containing AUG codons located in suboptimal sequence contexts, and thus undergo translation via leaky scanning. Structure-dependent pausing of the preinitiation complexes provides more time for the recognition of AUG codons in an unfavorable context. Whether this is a general mechanism remains to be determined, however, analysis of the predicted secondary structures downstream of initiation codons suggests that this may be the case [26]. The structural context of the AUG codon can modulate translation efficiency [27]. Coding sequences can also participate in the folding of the 5′ untranslated regions that modulate RNA stability [28,29]. However, coding sequence contributions to translation initiation remain understudied since functional and structural characterization is usually conducted on isolated 5′ UTR sequences. 

We set out to characterize how p22 translation is initiated. Our work suggests that both AUG codons can be utilized but AUG1 is used preferentially and translation efficiency strongly depends on the Ty1i RNA structure. Features stimulating p22 translation are context dependent as revealed by specific structures in Ty1i mRNA that are absent in full length genomic mRNA. The 5′ UTR of p22 mRNA interacts with the coding region and destabilization at the secondary or 3D structural levels results in a decrease in p22 translation. Also, our data supports the idea that protein factors such as Gag interact with a structural motif in Ty1i RNA to modulate its stability and translation. 

## 2. Materials and Methods

### 2.1. Preparation of the RNA Constructs for Structure Probing Experiments and In Vitro Translation Assays

All DNA templates for secondary structure probing experiments and in vitro translation were amplified from plasmid pBDG433, which contains transcribed sequences of Ty1-H3 subcloned into the riboprobe vector pSP64 (Promega, Madison, WI, USA). Forward and reverse primers are listed in Table S1. Each construct was confirmed by DNA sequencing. In vitro transcription reactions were performed using MEGAscript or MEGAshortscript T7 transcription kits (ThermoFisher, Waltham, MA, USA), as recommended by the manufacturer. RNA transcripts were purified using Direct-zol RNA MiniPrep Kit (Zymo Research, Irvine, CA, USA) and their integrity was monitored by formaldehyde agarose gel electrophoresis. Capped transcripts were synthesised in the presence of the ARCA Cap Analog (ThermoFisher). RNA used for native gel electrophoresis was [^32^P]-labelled at their 3′-ends with T4 RNA ligase (ThermoFisher) according to standard procedures.

### 2.2. Selective Acylation Analysed by Primer Extension (SHAPE)

The reaction mixture (100 μL) containing 20 pmol of RNA in SHAPE renaturation buffer (10 mM Tris-HCl pH 8.0, 100 mM KCl, 0.1 mM ethylenediaminetetraacetic acid (EDTA), pH 8.0) was heated at 95 °C for 3 min and placed on ice for 5 min. Fifty microliters of 3× SHAPE folding buffer (120 mM Tris-HCl pH 8.0, 600 mM KCl, 1.5 mM EDTA pH 8.0, 15 mM MgCl_2_) was added and samples were incubated for 30 min at 37 °C. Folded RNA was separated equally into two reactions and mixed with the 20 mM N-methylisatoic anhydride (NMIA) in dimethyl sulfoxide (DMSO) (2 mM final concentration of NMIA) or DMSO alone. Both reactions were incubated for 45 min at 37 °C followed by purification of RNA using Direct-zol RNA MiniPrep Kit.

### 2.3. DMS Modification

RNA (20 pmol in 50 μL) was refolded using the same conditions as those employed in the SHAPE experiments, then divided equally into two 24 μL reactions. Refolded RNA samples were mixed with 1 μL of dimethyl sulphate (DMS) in ethanol (0.5% final concentration) or ethanol alone. Both reactions were incubated 1 min at room temperature and mixed with 475 μL of stop solution (200 mM sodium acetate, 4.8 M β-mercaptoethanol). RNA was purified using Direct-zol RNA MiniPrep Kit immediately after stopping the reaction.

### 2.4. Hydroxyl Radical Probing

RNA samples (10 pmol) were refolded by heating at 95 °C for 2 min in water followed by incubation at 25 °C for 5 min. Next, 3× SHAPE folding buffer was added and the reaction was incubated for 25 min at 37 °C, then diluted 20× with 20 mM Tris-HCl pH 8.0. To initiate the production of hydroxyl radicals, 1.5 μL of 2.5 mM (NH_4_)Fe(SO_4_)_2_, 50 mM sodium ascorbate, 1.5% H_2_O_2_ and 2.75 mM EDTA were applied separately to the wall of the tube followed by centrifugation. Six microliters of water were added to the control reaction. Reactions were incubated for 10 s at room temperature, then quenched by the addition of thiourea and EDTA to final concentrations of 20 mM and 40 mM, respectively. RNA was recovered using Direct-zol RNA MiniPrep Kit.

### 2.5. Reverse Transcription and Data Processing

A reaction containing 2–5 pmol RNA, 10 pmol of fluorescently labelled primer PR5 or PR6 (Table S1) (Cy5 (+reagent) or Cy5.5 (control reaction)) and 0.1 mM EDTA pH 8.0 was incubated at 95 °C for 3 min, 37 °C for 10 min and 55 °C for 2 min, and then reverse transcribed at 50 °C for 45 min using Superscript III Reverse Transcriptase (ThermoFisher) as described previously [30]. Sequencing reactions were carried out using primers fluorescently labelled with LicorIR-800 (ddT) or WellRed D2 (ddA) and a Thermo Sequenase Cycle Sequencing Kit, according to the manufacturer’s protocol (Affymetrix, Santa Clara, CA, USA). Reverse transcription reactions and sequencing ladders were purified using ZR DNA Sequencing Clean-up Kit (ZymoResearch). cDNA samples were analysed on a GenomeLab GeXP Analysis System (Beckman–Coulter, Brea, CA, USA). Raw data were processed as described [31]. At least four repetitions were obtained for each reaction.

### 2.6. In Vitro Translation

In vitro translation experiments were carried out using wheat germ extract (WGE) as recommended by the manufacturer (Promega). The reaction mixture containing 12.5 μL of WGE lysate, 80 μM amino acid mixture minus methionine, 1.25 μL of [^35^S]-labelled methionine (1000 Ci/mmol) (Hartmann Analytic, Braunschweig, Germany), 79 mM potassium acetate, 20 units of ribonuclease inhibitor (ThermoFisher) and 1 pmol of refolded capped or uncapped RNA in the final volume of 25 μL was incubated for 1 hour at 25 °C. Translation products were resolved on sodium dodecyl sulphate (SDS)-polyacrylamide gels followed by radioisotope imaging using a FLA 5100 image analyser (Fuji, Minato, Tokyo, Japan). Bands intensities were analysed using MultiGauge software (Fuji). At least three repetitions were obtained for each in vitro translation reaction.

### 2.7. Native Gel Electrophoresis

[^32^P]-labelled RNA was refolded in SHAPE renaturation buffer by heating at 95 °C for 5 min and 4 °C for 5 min. SHAPE folding buffer contained increasing MgCl_2_ concentrations ranging from 0.1–10 mM. The reaction mixture (15 μL) was incubated at 37 °C for 25 min following the addition of 1.5 μL of 25% ficoll. Samples were analysed by native polyacrylamide gel electrophoresis using 12% gels in 0.5× TB at 4 °C. Electrophoresis was carried out at a gel temperature of 4 °C (DNApointer, Biovectis, Warsaw, Poland) [32]. Gels were dried, exposed to a phosphorimager screen, and scanned using FLA 5100 image analyser.

### 2.8. Ty1 Gag Expression and Purification

A Ty1 Gag-p45-GST fusion protein was expressed in *Escherichia coli* (*E. coli)* strain BL21(DE3)pLysS (Invitrogen, Carlsbad, CA, USA). Six liters of cells were grown in Luria-Bertani (LB) medium containing 50 μg/mL ampicillin and 34 μg/mL chloramphenicol at 28 °C to an OD_600_ of 0.7. Prior to isopropyl β-D-1-thiogalactopyranoside (IPTG) induction, cells were incubated for 30 min at 18 °C. Following the addition of IPTG (0.8 mM), the culture was induced at 18 °C overnight. Cells were pelleted by centrifugation at 4000 g for 10 min at 4 °C and resuspended in lysis buffer (50 mM Tris-HCl pH 8.0, 1 M NaCl, 10 mM β-mercaptoethanol, 2.5 mM DTT, 0.1 mM ZnCl_2_, 0.5 mg/mL lysozyme, and protease inhibitor (Roche, Basel, Switzerland)). The cell suspension was sonicated 40 × 2 s on ice with a 30 s pause after each pulse. Debris was removed by centrifugation at 20,000 g for 20 min at 4 °C. Nucleic acids were precipitated using 0.45% polyethyleneimine and pelleted by centrifugation at 30,000 g for 30 min at 4 °C. The supernatant was mixed with 1.5–2 mL of Glutathione Sepharose 4B (GE Healthcare, Little Chalfont, UK) and incubated for 1 h at 4 °C with gentle agitation followed by centrifugation at 700 g for 5 min. The Glutathione Sepharose beads were loaded onto a column and washed with 10 column volumes (10 mL/wash) of wash buffer (50 mM Tris-HCl pH 8.0, 1 M NaCl, 10 mM β-mercaptoethanol, 2.5 mM DTT, 0.1 mM ZnCl_2_). The glutathione S-transferase (GST) tag was removed by thrombin cleavage (GE Healthcare) at 4 °C for 12 h with gentle agitation. Ty1 Gag p45 was eluted using wash buffer, concentrated with centrifugal filtration (Millipore, Billerica, MA, USA), aliquoted and stored at −80 °C.

### 2.9. Filter Binding Assay

Reactions were performed in binding buffer (50 mM Tris-HCl pH 7.5, 40 mM KCl, 2 mM MgCl_2_, 0.01% Triton X-100) containing different concentrations of NaCl (50, 100, 150, 200, 250, 500 mM). [^32^P]-labeled domain I of Ty1i RNA (0.2 nM) was incubated for 4 min at 95 °C without magnesium ions and Triton X-100, and slowly cooled to 37 °C. MgCl_2_ and Triton X-100 were added following incubation for 10 min at 37 °C. Ty1 Gag protein solutions were prepared by sequential two-fold dilution of Gag in binding buffer. The binding reaction was initiated by mixing equal volumes of RNA and Gag protein in a microplate (final concentration of RNA was 0.1 nM). The reactions were incubated for 15 min at 24 °C, filtered and washed with 2 × 200 μL binding buffer containing 50 mM NaCl. A 96-well dot-blot (Minifold, Whatman, Maidstone, UK) was used with nitrocellulose (Protran, Whatman, Maidstone, UK) on top and charged nylon (Hybond N+, GE Healthcare) membranes on the bottom. Prior to use, both membranes were soaked in binding buffer containing 50 mM NaCl. After filtration, membranes were dried and exposed to a phosphoimager screen. Data were fitted to the Hill equation using Origin 8.5 software (OriginLab, Northampton, MA, USA). 

### 2.10. H1Δ Plasmid and Yeast Strains

The H1Δ deletion (T1015 - A1035) was generated by overlap PCR using flanking oligonucleotides Ty335F (5′-TGGTAGCGCCTGTGCTTCGGTTAC-3′) and RP1 (5′-ATAGTCAATAGCACTAGACC-3′), and overlapping oligonucleotides B (5′-GAAAGAATTTTCATGATAGGATGTCTTTGACCCAGGTAGGTAG-3′) and C (5′-GGTCAAAGACATCCTATCATGAAAATTCTTTCCAAAAGTATTGAAAAAA-3′). Wild-type pGPOLΔ (pBDG1130) [14] was used as the template for PCR. Nucleotide sequences correspond to the reference Ty1-H3 element (GenBank M10876.1). The H1**Δ** PCR product was cloned into pGPOLΔ using XhoI and BglII. The resulting plasmid pBAS47 is denoted as H1**Δ**. The H1**Δ** insert in pBAS47 was verified by DNA sequencing. Plasmids pBDG1130 and pBAS47 were transformed into the following strains: DG2196 (1 Ty1) [13] to generate DG2374 and YAS89, and DG3582 (0 Ty1) [14] to generate YAS85 and YAS87, respectively.

### 2.11. Northern and Western Blotting

Yeast cultures for total cellular RNA and protein extraction were grown in SC-Ura + 2% glucose medium at 22 °C for 24 h. RNA was extracted using the MasterPure Yeast RNA purification kit (Epicenter Biotechnologies, Madison, WI, USA) [14]. For each strain, 8 μg total RNA was separated on a 1.2% formaldehyde-agarose gel and subjected to Northern blot analysis using [^32^P]-labeled riboprobes corresponding to Ty1 nucleotides 1266–1601 and *ACT1*, followed by phosphorimaging using a STORM 840 phosphorimager and ImageQuant software (GE Healthcare) [13]. Protein isolation and Western blot analysis to detect p22 was performed as described previously [14]. A rabbit polyclonal antisera against Pgk1 (kindly provided by Jeremy Thorner) was used at a 1:100,000 dilution. Immune complexes were detected with enhanced chemiluminescence (ECL) reagent (GE Healthcare). The amount of p22 relative to Pgk1 was estimated by densitometry using Quantity One software (Bio-Rad). Northern and Western analyses using the 0 Ty1 and 1 Ty1 strains containing pGPOLΔ or pH1Δ were repeated twice and representative results are presented. Also, independent Western analyses using the 0 Ty1 strain containing pGPOLΔ or pH1Δ were repeated three more times.

Ty1*his3-AI* mobility frequencies were determined as described previously [13,33]. Briefly, a single colony was resuspended in 1 mL water and four; 1 mL SC-Ura cultures were inoculated with 5 μL of cell suspension. Quadruplicate cultures for each strain were grown at 22 °C for three days. Cells were pelleted, resuspended in 1 mL water, and dilutions spread on SC-Ura and SC-Ura-His plates were incubated at 30 °C for 4 days. The frequency of Ty1*his3-AI* mobility was calculated by the number of His^+^ Ura^+^ colonies/the number of Ura^+^ colonies per mL of culture.

### 2.12. RNA 3D Structure Prediction

Structure prediction experiments were performed by RNAComposer [34] webserver [35]. The AUG1AUG2 RNA domain I sequence: GGGUCAAAGACAUCCUAUCCGUUGAUUAUACGGAUAUCAUGAAAAUUCUUUCCAAAAGUAUUGAAAAAAUGCAAUCUGAUACCC and secondary structure topology in dot bracket notation: ((((...((((((..(((((((.......)))))))............(((.((.......)).))).)))...)))...)))) were used as input data. The 3-way junction of domain I of AUG1AUG2 RNA was generated by RNAComposer, therefore, it was substituted by the elements introduced by the user. This element was chosen from RNA structures deposited in Research Collaboratory for Structural Bioinformatics (RCSB) Protein Data Bank (PDB) database following the criteria of the highest homology of secondary structure topology and sequence. More than 10 batches with different three-way junction structures were run. Ten models were generated for every batch. The resulting models were clustered based on the agreement with the hydroxyl radical cleavage data and the energy. Hydroxyl radical cleavage reactivity indexes from experiments were compared with indexes denoting atomic crowding around phosphorus at the corresponding nucleotide residue. The models with correct energy [36] and the best similarity were accepted.

## 3. Results and Discussion

### 3.1. Both AUG Codons in Ty1i RNA Can Be Recognized for Translation Initiation

Our previous results demonstrated that p22 translation can be initiated from AUG1 and AUG2 codons and is strictly cap-dependent. Also, either AUG1 or AUG2 can function to initiate translation when the other is mutated [14]. However, a number of questions remain unanswered: (i) Are both AUGs active for translation when present in the same RNA? (ii) Or is one codon translated preferentially? (iii) Does leaky scanning account for p22 synthesis from AUG2? Moreover, deleting the 5′ UTR or mutating AUG1 or AUG2 decreases the level of p22 in vivo. For AUG1 and AUG2 codon mutants, the decrease in the p22 level is significantly larger than expected considering that one AUG codon is still present. These results suggest that the structure of the 5′ terminal part of Ty1i RNA may influence p22 translation. 

**Figure 1:**
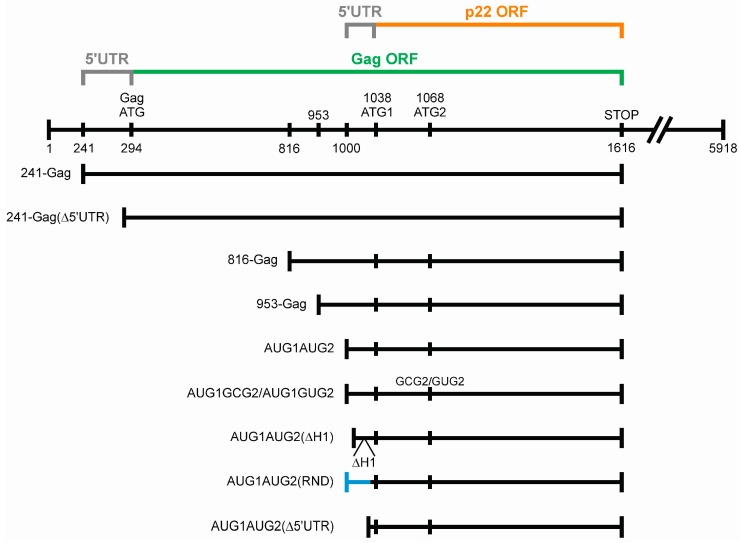
RNA constructs used in this study. Nucleotide positions correspond to the Ty1H3 DNA sequence (GenBank accession M18706.1) [15]. 5′ UTR: 5′ untranslated region, ORF: open reading frame.

**Figure 2:**
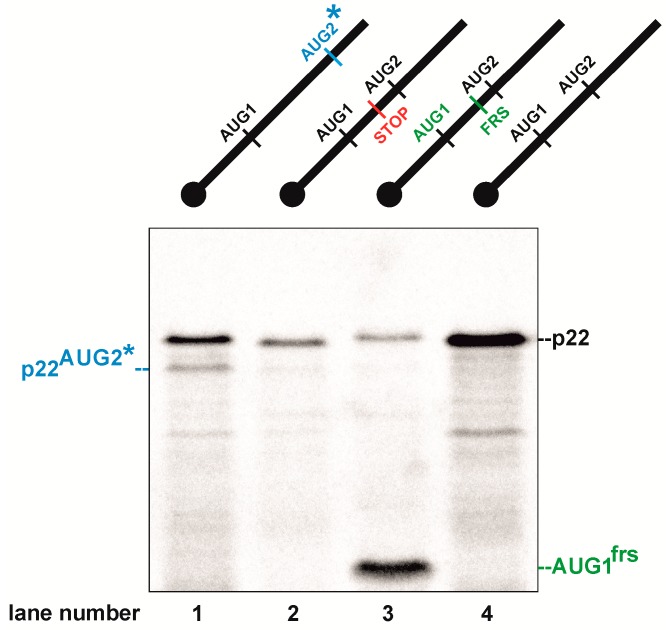
In vitro translation of Ty1i RNA and its derivatives in wheat germ extract. In vitro transcribed, capped RNA AUG1AUG2*, AUG1^stop^AUG2, AUG1^frs^AUG2 and AUG1AUG2 were translated in the presence of ^35^S-methionine followed by electrophoresis and autoradiography. Schematic representation of RNA molecules is shown above the gel (see text for details).

Translational activity of both AUG codons could be beneficial and contribute to the evolutionary diversification of p22. To gain insights into translation from AUG1 and AUG2 in Ty1i RNA, we performed in vitro translation assays using three derivatives of AUG1AUG2 RNA [14]. AUG1AUG2 RNA started at nt 1000 of Ty1, comprised the 5′ UTR and p22 open reading frame (ORF), and ended with a natural stop codon (Figure 1). The difference between p22 proteins translated from AUG1 and AUG2 is only 10 amino acid residues (30 nt). Such a small size difference makes the two proteins difficult to separate by gel electrophoresis and obscures simultaneous analysis of the translation levels from both AUGs. To overcome this difficulty, we synthesized AUG1AUG2* RNA in which AUG2 (including its Kozak context) is 30 nucleotides downstream of the original AUG2, and introduced a GCG alanine codon in place of AUG2 (Figure 2). This modification increased the distance between AUG1 and AUG2* to 60 nt (20 amino acids), which allowed separation of the two translation products. A frameshift mutation (insertion of AU between U1050 and C1051) was introduced in AUG1^frs^AUG2 RNA (Figure 2). In this case, translation from AUG1 occurred out of frame in relation to AUG2 and resulted in the synthesis of a 49-amino acid peptide. Translation of the AUG1AUG2* and AUG1^frs^AUG2 RNAs allowed us to determine if both AUGs were recognized for translation. The third RNA, AUG1^stop^AUG2, contained an insertion of a single U between U1060 and U1061, which introduced a premature stop codon following translation from AUG1 (Figure 2). This RNA mutation was designed to help determine the level of p22 translated from AUG2. Each construct was also designed to avoid the introduction of rare codons that could obscure translation. 

AUG1AUG2* RNA was translated into two products: p22^AUG1^ synthesized from the natural AUG1 and the shorter protein p22^AUG2*^ (Figure 2, lane 1). p22^AUG1^/ p22^AUG2*^ were synthesized in a ratio of 5:1, which indicates that AUG1 is the main site of p22 translation initiation in AUG1AUG2* RNA. However, the translational activity of AUG1AUG2* RNA decreased 75% when compared with wild-type AUG1AUG2 RNA. Two proteins were also translated from the AUG1^frs^AUG2 RNA: a faster migrating out of frame AUG1^frs^ peptide, and p22^AUG2^, which originated from the natural AUG2 triplet (Figure 2, lane 3). AUG1^frs^/p22^AUG2^ were synthesized in a ratio of 6:1, which is similar to AUG1AUG2*, and confirms that AUG1 is utilized preferentially for p22 initiation in these two RNAs. As expected, p22^AUG2^ that initiated from AUG2 was detected with AUG1^STOP^AUG2 RNA (Figure 2, lane 2). The level of AUG2-initiated p22 was low but comparable between different constructs.

Taken together, the results of in vitro translation show that both AUG codons present in Ty1i RNA can be actively translated and AUG1 is preferentially utilized to initiate p22 synthesis. Our results also suggest that leaky scanning is the most likely mechanism for p22 translation from AUG2. Experimental support for leaky scanning is illustrated by the decrease of AUG2 translation levels from AUG1AUG2* and AUG1^frs^AUG2 RNAs (having both p22 AUG codons) in comparison to GCG1AUG2 RNA mutant where only AUG2 is present [14]. Moreover, the translational activity of AUG1AUG2* and AUG1^frs^AUG2 RNAs was significantly lower when compared to wild-type AUG1AUG2 RNA. These results raise the possibility that AUG1AUG2* and AUG1^frs^AUG2 RNAs affect the structure of the 5′ UTR of Ty1i RNA, leading to translation inhibition, and that the 5′ UTR may also regulate the production of p22.

### 3.2. The 5′ UTR of mRNA Interacts with the p22 Coding Region

Significant loss of translational activity from AUG1 in AUG1GCG2 [14] (Figure 1), AUG1AUG2* and AUG1^frs^AUG2 RNAs suggests that the structure of the region containing AUG1 and AUG2 is important for p22 translation. Therefore, we performed selective 2′-hydroxyl acylation analyzed by primer extension (SHAPE) [37] on the 5′ terminal region of Ty1i RNA to examine its secondary structure. N-methylisatoic anhydride (NMIA) preferentially modifies 2′OH groups of single-stranded and flexible nucleotides in RNA. Primer extension of fluorescently labeled primers by reverse transcriptase is blocked at modified positions in RNA, and these truncated DNA products can be identified using capillary electrophoresis. Secondary RNA structures were obtained by computational analysis of the reverse transcription products. Secondary structure probing experiments were carried out on AUG1AUG2 RNA that was used in the in vitro translation studies. This ~630 nt long RNA contained the 5′ UTR of Ty1i RNA (37 nt) and coding sequence of p22 (Figure 1). 

**Figure 3:**
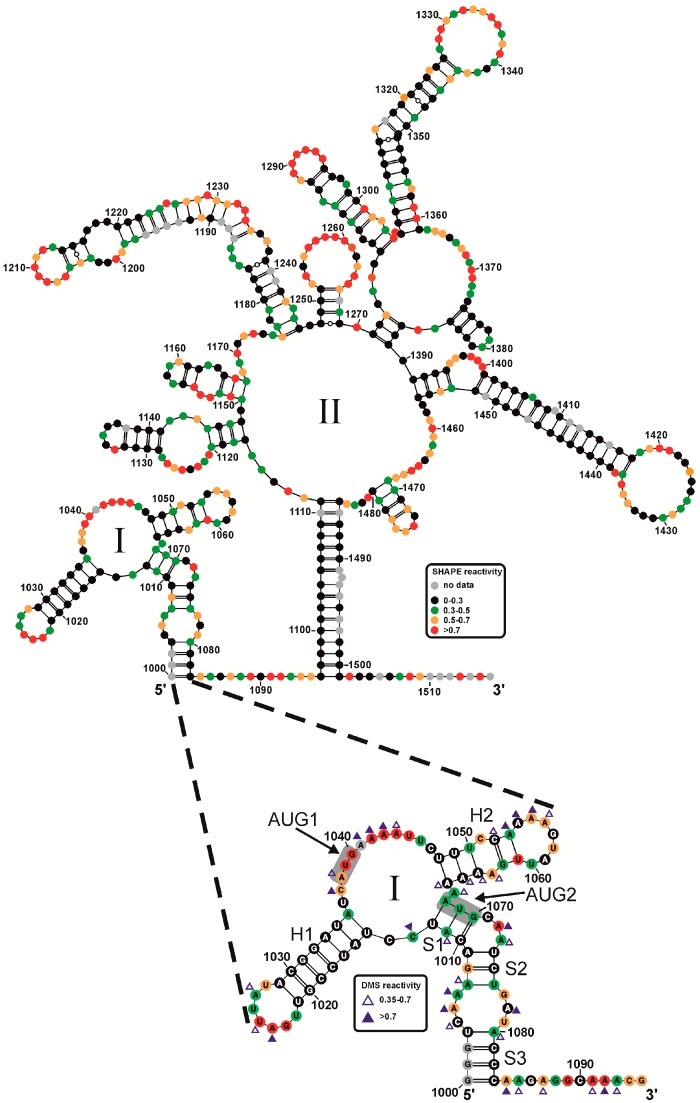
Secondary structure model of the 5′ terminal segment of Ty1i RNA (upper panel) and a detailed view of domain I (bottom panel) predicted by the *RNAstructure* software with experimental constraints [38]. Nucleotides are coloured according to their selective 2′-hydroxyl acylation analyzed by primer extension (SHAPE) reactivity (black, green, orange, red). The blue triangles (filled and open) represent dimethyl sulfate (DMS) modifications.

Figure 3 shows a secondary structure model of the 5′ terminal part of the Ty1i RNA [15] predicted using the *RNAstructure* software [38,39] which incorporates experimental constraints from SHAPE mapping. 

Our results suggest that Ty1i RNA folds into two major domains. The smaller domain I (G1000–1083) and larger domain II (A1096–U1501) were connected by a 12nt-long single-stranded region (A1084–G1095). 

Interestingly, domain I included the Ty1i 5′ UTR and p22 coding sequence, and contained both p22 initiation codons (Figure 3). This structure is organized by the interaction of the proximal part of the 5′ UTR (G1000–U1012) with a stretch of coding sequence (A1068–C1083; stems S1–S3). Also, two hairpin structures were present. Hairpin H1 (U1015–A1035) was composed of residues from the 5′ UTR while hairpin H2 (U1048–A1066) contained nucleotides from the coding sequence. A three-way junction connected hairpins H1, H2 and stem S1. 

The data from SHAPE probing support the predicted structure of domain I. Nucleotides within single-stranded regions were reactive towards the SHAPE reagent, including apical loops of both hairpins, internal loops, bulges and mismatches. The presented structure was also supported by dimethyl sulfate (DMS) probing. DMS methylates N1 of adenosines and N3 of cytidines that have an accessible Watson–Crick edge of the base rings [40]. In our structure, almost every A and C residue predicted to be single-stranded was susceptible to DMS methylation. However, some nucleotides in the hairpin H2 stem were methylated moderately by DMS but remained unreactive towards NMIA. These results support the idea that the C1052 and A1064-A1066 hairpin region is constrained by non-standard base pairing.

Interestingly, domain I contained both p22 initiation codons localized in different structural contexts (Figure 3). AUG1 constituted part of the 12nt-long single-stranded region U1036–C1047 while AUG2 was embedded in the double-stranded S1 stem that was formed by interactions of nts 1068–1070 with the residues of the 5′ UTR (C1010–U1012). The S1 stem may be thermodynamically unstable since the AUG2 triplet was somewhat reactive against NMIA.

Domain II folded into a large multibranched structure (Figure 3) organized by extensive pairing between A1096–C1111 and G1485–U1501. As a result, a 16 bp duplex region was formed. Domain II contained a complex junction that connected six simple hairpin structures and one branched region in a three-way junction motif. The majority of the single stranded regions were well mapped by NMIA. Importantly, the NMIA modification pattern of nucleotides spanning domain II in AUG1AUG2 RNA was very similar to the same region mapped inside VLPs using in virio SHAPE [30] (please note that the numbering herein corresponds to the complete Ty1H3 element while the numbering in reference [30] corresponds to Ty1 genomic RNA [30]). This result suggests that our in vitro folding conditions recapitulate the native structure of Ty1 RNA.

### 3.3. The 3D Structural Integrity of Domain I Affects p22 Translation 

We reported that the combined level of p22 synthesized from AUG1GCG2 and GCG1AUG2 RNA constitutes only 30% of that obtained from wild-type AUG1AUG2 RNA [14]. Secondary structure probing of AUG1AUG2 RNA revealed that both p22 initiation codons were located within the same domain. Thus, mutation of AUG1 or AUG2 could cause structural perturbations that inhibit p22 translation. Since the in vitro translation results (Figure 2) identified AUG1 as a main translation initiation site for p22 synthesis, we hypothesized that mutating AUG2 to GCG strongly inhibited translation from AUG1 due to changing the structural context of AUG1 in domain I. The AUG2 to GCG mutation also introduced a U–G wobble pair as well as A–C mismatch that could affect the double-stranded character of the S1 stem. 

**Figure 4:**
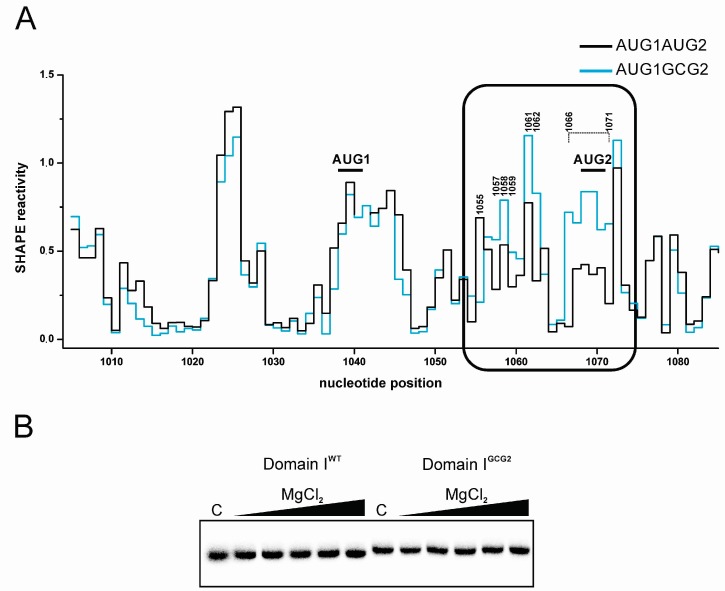
(**A**) SHAPE reactivity profile of the AUG1AUG2 (black) and AUG1GCG2 (blue) domain I as a function of nucleotide position. Nucleotides that changed their reactivity in domain I^GCG2^ are indicated. (**B**) Native gel electrophoresis of the [^32^P]-labeled wild-type and mutated domain I of Ty1i RNA at increasing concentrations of MgCl_2_. C: control reaction without MgCl_2_. WT: wild type.

To determine if the GCG mutation altered the structure of domain I, we performed secondary structure probing of AUG1GCG2 RNA using SHAPE. Although the overall reactivity pattern of the AUG1GCG2 RNA was preserved (Figure 4A), the region of domain I containing the GCG mutation (A1066–A1071) became highly reactive. This alteration suggests that the mutant RNA residues in the S1 stem are single-stranded or this region is highly unstable. Additionally, several nucleotides in hairpin H2 displayed a different pattern of reactivity: G1057–A1059, U1061 and G1062 exhibited higher reactivity while A1055 had decreased reactivity. Surprisingly, the structural motifs in the neighborhood of AUG1 remained essentially the same in wild type and mutant AUG1AUG2 RNA. Moreover, the GCG mutation did not change the secondary structure of domain II (data not shown). Overall, our data suggests that the GCG mutation disrupts the three-dimensional structure of domain I, which in turn inhibits the translation of p22 from AUG1. 

Our model suggests that a three-way junction element (Figure 3) governs the special organization of domain I. By disrupting the S1 stem, the GCG mutation might change the topology and relative positioning of the H1 and H2 hairpins. Changes in the three-dimensional structure of RNA molecules can be monitored by native polyacrylamide gel electrophoresis [41]. Therefore, we subjected the isolated domain I (nts G1000–C1083) containing the GCG mutation (domain I^GCG2^) along with the wild-type domain I to native gel electrophoresis (Figure 4B). We observed a slower mobility of domain I^GCG2^ RNA, which may reflect a change in the three-dimensional structure of domain I when compared with wild type. Migration of both wild-type and GCG mutated domain I remained unchanged at a higher concentration of Mg^2+^ ions, suggesting that this part of Ty1i RNA undergoes unimolecular folding [42].

The results obtained by native gel electrophoresis suggest that the double-stranded character of the S1 stem is an important factor stabilizing the three-dimensional structure of domain I. To help preserve the double-stranded character of stem S1, we mutated AUG2 to a GUG valine codon that changed only the first U–A pair to a U–G wobble pair (Figure 1). Secondary structure probing of AUG1GUG2 mutant RNA indicated that the S1 stem was slightly destabilized (Figure S1). Moreover, two residues directly upstream of the S1 stem (A1066 and A1067) were more reactive, suggesting an enhancement of local flexibility. A1066 and A1067 were also strongly modified in AUG1GCG2 mutant RNA. Some of the nucleotides in the H2 hairpin that changed their reactivity in AUG1GCG2 RNA behaved in a similar manner in AUG1GUG2 RNA. Higher reactivity of U1058 and A1062 as well as lack of reactivity of A1055 was detected. A1063 was also less reactive in AUG1GUG2 RNA when compared to wild type AUG1AUG2. Importantly, the structural context of AUG1 was preserved, which is similar to the AUG1GCG2 and AUG1GUG2 mutants. Taken together, our data suggest that the GUG2 mutation destabilized the S1 stem much less than the GCG2 mutation, and the structural integrity of the S1 stem and hairpin H2 are important determinants for the proper three-dimensional structure of domain I.

**Figure 5:**
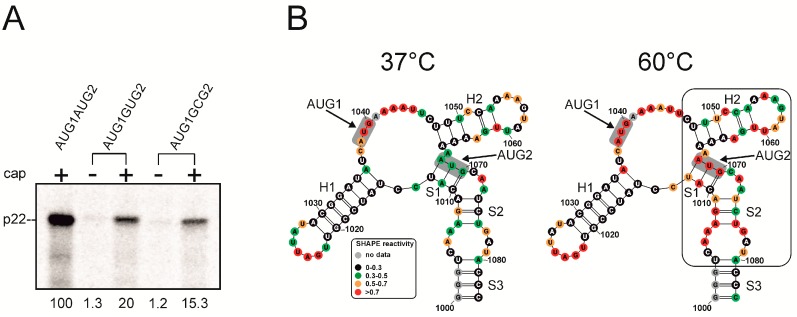
In vitro translation of AUG2 mutational variants of Ty1i RNA and melting profile of AUG1AUG2 RNA. (**A**) In vitro transcribed capped or uncapped transcripts were translated using wheat germ extract in the presence of ^35^S-methionine. Calculated translation activity (in relation to the capped AUG1AUG2 RNA) is shown below the gel. (**B**) Melting of AUG1AUG2 RNA followed by SHAPE at 37 °C and 60 °C. Nucleotides are coloured according to their reactivity (black, green, orange, red). The segment of domain I with the strongest changes at 60 °C is boxed.

Mutation of AUG2 to GCG2 markedly inhibits p22 translation (Figure 2) [14]. Since we determined that the GUG2 mutation had a less profound effect on the domain I secondary structure, we analyzed the translational activity of capped and uncapped AUG1GUG2 RNA along with AUG1GCG2 and AUG1AUG2 RNA in vitro (Figure 5A). In agreement with our previous study [14], p22 translation from AUG1GCG2 RNA was inhibited to ~15% of the initial value calculated for AUG1AUG2 RNA. Interestingly, the translation of p22 from AUG1GUG2 RNA was also inhibited to ~20% when compared with wild type RNA. These results further extend our finding that the structural integrity of the domain I of Ty1i RNA contributes significantly to the efficient translation of the p22 from AUG1, and even small structural changes impair translation in vitro.

Placement of the initiation codon in thermodynamically stable secondary structures can decrease its translational activity [43]. However, the calculated thermodynamic stability [44] of domain I in wild-type Ty1i RNA was only −25.2 kcal/mol, and AUG1 was predicted to reside in a long single-stranded region (Figure 3). To assess the thermodynamic stability of the 5′ terminal segment of Ty1i RNA, we determined the reactivity profile of AUG1AUG2 RNA by SHAPE mapping at different temperatures (Figure 5B). SHAPE analysis at 37 °C and 60 °C identified residues within domain I that changed their reactivity at 60 °C. Interestingly, the most pronounced effects were observed in the regions prone to destabilization in RNA mutants AUG1GCG2 and AUG1GUG2 (Figure 4 and Supplementary Figure S1). At 60 °C, the nucleotide stretch A1067–G1077 (including AUG2) as well as the opposite strand A1005–C1013 became highly reactive, suggesting that the strands dissociate. Also, several residues located in the hairpin H2 stem (U1049–C1052) and in the apical loop (A1059–U1061) were altered, suggesting that the region containing AUG2 and hairpin H2 is less stable than other parts of domain I.

### 3.4. Structure of Domain I Specific for Ty1i RNA Stimulates p22 Translation

**Figure 6:**
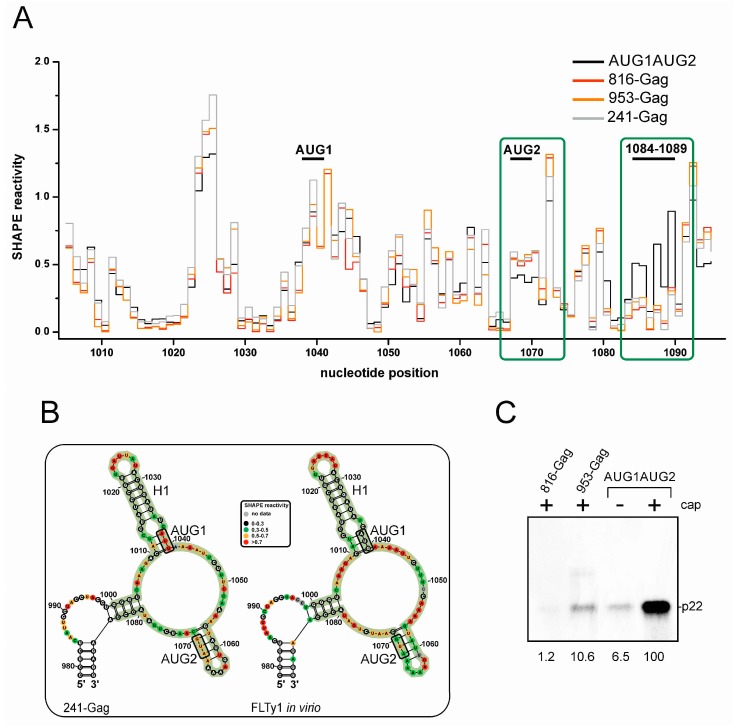
Secondary structure probing and the in vitro translation of 241-Gag RNA and its derivatives. (**A**) Reactivity plot of nucleotides spanning domain I in AUG1AUG2 RNA (black), 816-Gag RNA (red), 953-Gag RNA (orange) and 241-Gag RNA (grey). Regions showing consistent differences in reactivity are boxed (green). (**B**) Comparison of the secondary structure models of domain I obtained in vitro for 241-Gag RNA (left) and full-length genomic Ty1 RNA within virus-like particles (VLPs) (in virio conditions; right). Nucleotides that cover domain I in Ty1i RNA are marked (green background). p22 initiation codons and the H1 hairpin are also highlighted. (**C**) In vitro translation of sequential variants of Ty1 genomic RNA. Capped or uncapped transcripts were translated in wheat germ extract in the presence of the ^35^S-methionine. Quantitation of the translation products is shown below the gel.

In vitro translation and secondary structure probing of the 5′ terminal part of wild-type and mutant Ty1i transcripts suggest that domain I plays an important role in the efficient translation of p22 from AUG1. Previous results show that p22 is not translated from the full-length genomic RNA [15]. These findings motivated us to ask whether the structure of domain I was stable in the context of a larger RNA that more closely resembles Ty1 genomic RNA. To this end, we analyzed a ~1400 nt RNA (nts 241–999 using the coordinates of the complete Ty1H3 element), termed 241-Gag RNA, that began from the first nucleotide of the genomic Ty1 RNA, and included the structured 5′ UTR [30,45] and Gag coding sequence (Figure 1). Comparison of SHAPE reactivity profiles of 241-Gag and AUG1AUG2 RNAs revealed different modification patterns of domain I (Figure 6A). 

The reactivity of the region encompassing AUG2 (A1067–A1072) increased in 241-Gag RNA while the proximal part of the single-stranded region connecting domains I and II (A1084–G1089) lost accessibility to NMIA modification. The observed alterations suggest that domain I and the neighboring regions fold differently when the 5′-terminal sequence of genomic RNA is present in the transcript. 

The secondary structure of the full-length Ty1 RNA has been determined inside virus-like particles (VLPs) by in virio SHAPE analysis [30]. In the proposed structure for Ty1 genomic RNA, the sequence encompassing domain I is folded differently than in Ty1i RNA (Figure 6B). Interactions between C979–U983 and A1085–G1089 extended domain I in the full-length transcript. Moreover, the structural context of the p22 initiation codons differed significantly. Unlike their context in Ty1i RNA, AUG1 was fully paired with the C1010–U1012 in full-length Ty1 RNA. Interestingly, the C1010–U1012 region was also paired but with the AUG2 codon forming the S1 stem in Ty1i RNA (Figure 3). AUG2 was localized in the stem of a predicted unstable hairpin G1057–C1071. The only common structural element within the region encompassing domain I in the full-length Ty1 and Ty1i RNAs was hairpin H1, suggesting that hairpin H1 folds independently of the structural elements present in its vicinity.

Importantly, comparing the reactivity profiles of 241-Gag and full-length Ty1 RNA [30] revealed that domain I folding was similar (Figure 6B). The main difference was AUG1 reactivity, which was high in 241-Gag RNA and low in full-length Ty1 RNA. This difference suggests that the cellular environment in this region, such as the presence of the Gag chaperone, folds the RNA into a more stable structure. 

The distinct structure of the region encompassing domain I in the full-length Ty1 RNA raised a question concerning how domain I might influence p22 translation. The initiation of p22 synthesis from the 241-Gag RNA is unlikely to occur, which raises the possibility that p22 synthesis requires a specific structure of domain I in Ty1i RNA [14]. The presence of the Gag AUG initiation codon as well as seven internal in-frame AUG codons before encountering AUG1 would preclude migration of the preinitiation complexes downstream of the AUG1 and AUG2 initiation codons. Additionally, the 5′ UTR of Ty1i RNA in the 241-Gag RNA would be extended to over 700 nucleotides, which could greatly affect the scanning mechanism. To address whether a specific structure of the domain I of Ty1i RNA is necessary for the efficient translation of p22, we synthesized 816-Gag and 953-Gag RNAs (Figure 1). Both RNA molecules were designed to possess full-length folding of domain I, which is supported by their similar reactivity profile when compared to 241-Gag RNA (Figure 6A). The 816-Gag and 953-Gag RNAs were translated in vitro in wheat germ extract (Figure 6C). We observed that p22 protein was poorly translated from both RNA molecules and could be detected only when capped transcripts were used. Low levels of translation from extended Ty1 transcripts with the full-length-like folding of the region 1000–1083 suggests that the structure of the domain I observed in Ty1i RNA specifically stimulates p22 translation from AUG1.

### 3.5. The Ty1i RNA 5′ UTR Stimulates p22 Translation

To further understand the role of the Ty1i 5′ UTR in p22 translation, we analyzed in vitro several mutant RNA constructs (Figure 1). In AUG1AUG2(Δ5′ UTR), 32 of 37 nucleotides of the 5′ UTR have been deleted while in AUG1AUG2(RND) the same sequence was replaced by 32 random nucleotides. In AUG1AUG2(ΔH1), the common structural element of full-length Ty1 and Ty1i RNA (hairpin H1) was deleted (nts 1015–1031). Also, all transcripts maintained an intact Kozak context adjacent to the AUG1 initiation codon. 

**Figure 7:**
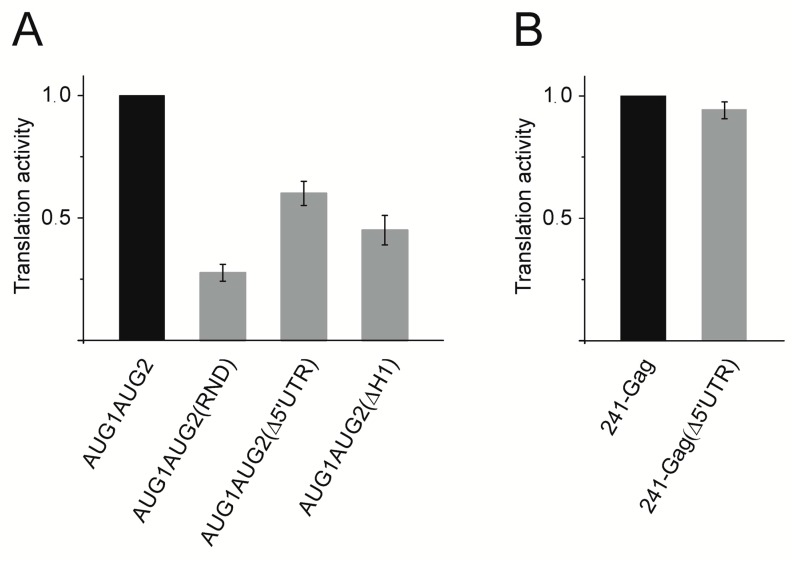
In vitro translation of the capped variants of the AUG1AUG2 RNA and 241-Gag RNA. Translational efficiency was normalized to the amount of the protein product synthesized from AUG1AUG2 RNA (**A**) or 241-Gag RNA (**B**).

We observed significant inhibition of p22 translation from all three RNA constructs (Figure 7A). Deleting the 5′ UTR inhibited p22 translation by 40% when compared to wild-type AUG1AUG2 RNA. These results suggest that the Ty1i 5′ UTR is required for efficient p22 synthesis. Since shortening the 5′ UTR to only six nucleotides could interfere with ribosome scanning [46,47,48], we analyzed 241-Gag(Δ5′ UTR) RNA possessing 5′ UTR that was also reduced to six nucleotides. However, the translation of Gag was unaffected (Figure 7B). This result suggests that the inhibitory effect observed for AUG1AUG2(Δ5′ UTR) may impair the structure of domain I. The important role of the 5′ UTR in p22 translation was also supported by the translation of AUG1AUG2(RND) and AUG1AUG2(ΔH1) RNAs. Despite having a 5′ UTR of the same length as wild-type, AUG1AUG2(RND) RNA displayed >70% inhibition in p22 translation. A 55% inhibition of p22 synthesis was also observed with AUG1AUG2(ΔH1) RNA. Taken together, our data suggest a stimulatory role for the Ty1i 5′ UTR in the translation of p22 due to its involvement in the folding of domain I.

### 3.6. Gag Interacts Specifically with Ty1i Domain I In Vitro

**Figure 8:**
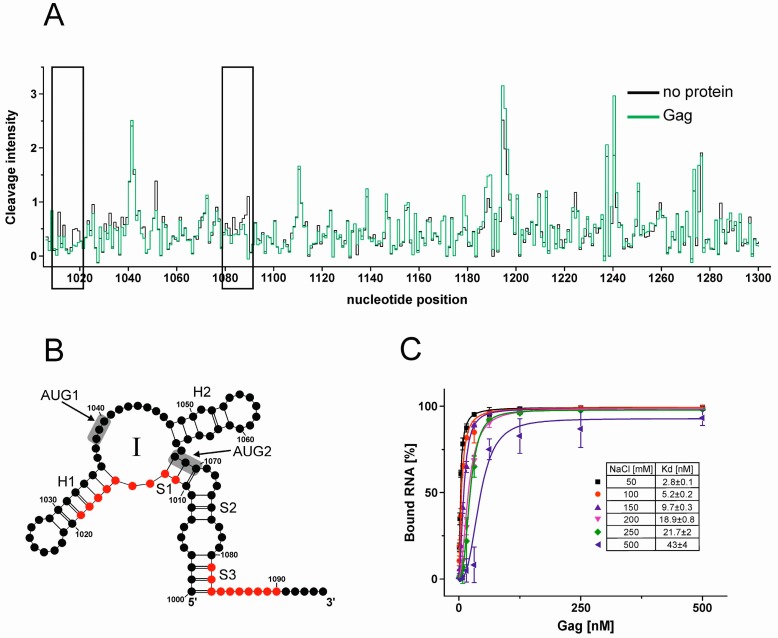
RNA binding properties of recombinant Ty1 Gag-p45. (**A**) Hydroxyl radical reactivity plots of protein free AUG1AUG2 RNA (black) in comparison with RNA probed in the presence of Gag (green). Regions showing consistent decreased reactivity over several nucleotides in the presence of Gag are boxed. (**B**) 2D structure model of Ty1i domain I with the positions protected from hydroxyl radical cleavage in the presence of the Ty1 Gag are indicated (red). (**C**) Filter-binding assay performed with Ty1i domain I RNA and Gag at different concentrations of NaCl (50–500 mM). The lines correspond to the best fit of the data. The error bars represent standard deviations. Kd: dissociation constant.

Translation initiation can be regulated not only by RNA structure but also by protein factors that interact with structural elements in mRNAs [19]. Since the amount of Gag and p22 determines the level of inhibition of Ty1 mobility [49], perhaps Gag modulates the efficiency and/or timing of p22 translation. Potential Gag binding sites in the 5′ terminal part of Ty1i RNA were detected by hydroxyl radical footprinting of AUG1AUG2 RNA complexed with recombinant Gag-p45 (Figure 8A). The protected sequences were identified by comparing the reactivity profiles of AUG1AUG2 RNA in the presence and absence of Gag. Only regions in domain I displayed decreased susceptibility to hydroxyl radical cleavage in the presence of Gag, including residues A1011–C1019 that comprise part of the S1 stem and the hairpin H1 stem. Another potential Gag binding site was localized in the p22 coding region (nts A1084–G1095) connecting domains I and II. In particular, C1081–C1090 was protected from the cleavage in the presence of Gag (Figure 8A,B). 

To further investigate the interaction between Gag and domain I, we calculated dissociation constants of RNA/protein complex formation using a double filter binding assay (Figure 8C). We used isolated domain I that was extended by the single-stranded stretch connecting domain I and II (RNA I^1000-1095^) to encompass both Gag binding sites. The calculated dissociation constant (Kd ~3 nM) suggests that there is a high affinity binding site for Gag in domain I. To examine whether Gag binding is specific, we determined the Kd with increasing concentrations of NaCl, which is often used to compete out non-specific RNA/protein interactions [31]. The Gag/domain I interaction was slightly affected in the 100–250 mM NaCl range and persisted even at 500 mM NaCl (Kd ~43 nM). Taken together, the results from chemical footprinting and filter binding suggest that the interaction between Gag and domain I is strong and highly specific. 

### 3.7. Deleting the Hairpin H1 Sequence Decreases Stability of Ty1i RNA In Vivo

**Figure 9:**
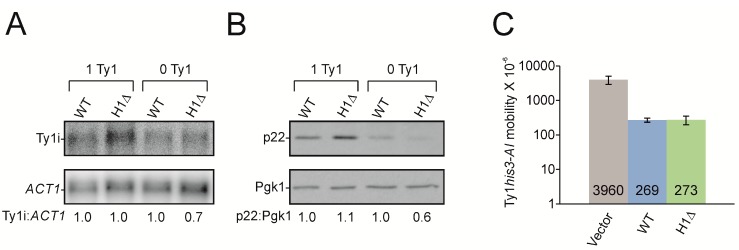
**Effect of the hairpin H1 deletion on Ty1i RNA, p22 protein expression and Ty1*his3-AI* mobility**. (**A**) Northern blotting of total RNA from the 1 Ty1 strain (DG2196) and 0 Ty1 strain (DG3582) containing either wild type (WT) pGPOLΔ or mutant pH1Δ plasmids. A [^32^P]-labeled Ty1 riboprobe (nt 1266 to 1601) was used to detect Ty1i RNA. *ACT1* mRNA served as a loading control. Below are Ty1i:*ACT1* ratios as determined by phosphorimaging. (**B**) Whole cell extracts from strains used in (A) were immunoblotted with p18 antiserum to detect p22. Pgk1 served as a loading control. p22:Pgk1 ratios were determined by densitometry. (**C**) Quantitative Ty1*his3-AI* mobility assayed in the 1 Ty1 strain containing one genomic Ty1*his3-AI* element and empty vector, WT, or H1Δ plasmids. All strains were grown in glucose containing medium to repress *GAL1*-promoted Ty1 expression. Bars denote standard deviation.

To investigate the effects of the H1 hairpin on Ty1i RNA and p22 expression in vivo as well as on Ty1 transposition, a mutated pGPOLΔ plasmid was constructed (pBAS47, termed H1Δ) that expresses Ty1i RNA lacking the H1 sequence (U1015–A1035) from the 5′ UTR (Figure 9). Wild type pGPOLΔ is a multicopy expression plasmid containing most of the Ty1 5′LTR and *GAG* that is driven by the *GAL1* promoter [15]. When yeast cells containing pGPOLΔ are grown in glucose media, *GAL1* promoted transcription of Ty1 is repressed. However, Ty1i RNA and p22 are still expressed from pGPOLΔ under glucose repression since Ty1i RNA is transcribed from internal initiation sites. 

We investigated the effect of H1Δ on Ty1i RNA level in a *S. paradoxus* strain with 1 chromosomal Ty1 element (DG2196; 1 Ty1) and the isogenic Ty1-less parent (DG3582; 0 Ty1) that contain WT pGPOLΔ or pH1Δ plasmids (Figure 9A). Northern blotting of total RNA from these strains showed no change in Ty1i RNA levels in the H1Δ mutant compared to the wild type (WT) plasmid in the 1 Ty1 strain. However, Ty1i H1Δ RNA levels decreased about 30% compared to WT Ty1i RNA in the 0 Ty1 strain (refer to Materials and Methods). These results suggest that the H1 hairpin may affect the stability of Ty1i RNA. In the 1 Ty1 strain, however, the defect in Ty1i H1Δ RNA stability was not evident. This may be due to additional Gag binding sites on Ty1i RNA that stabilize the transcript in the 1 Ty1 strain, as suggested by hydroxyl radical footprinting (Figure 8). Note that Gag binding sites C1081–C1090 remain intact in Ty1i H1Δ RNA and could function in vivo.

Total cell extracts from the same strains were subjected to Western analysis using an antiserum that detects p22 [14] (Figure 9B). The level of p22 remained about the same in the 1 Ty1 strain containing WT or H1Δ plasmids. In the 0 Ty1 strain, p22 decreased 43% (±12%) in the mutant pH1Δ when compared to WT pGPOLΔ. These results suggest that there is a correlation between p22 and Ty1i RNA levels (Figure 9A) in both strain backgrounds containing WT or H1Δ plasmids.

Finally, we asked if deleting the H1 hairpin from the Ty1i RNA affected Ty1 mobility (Figure 9C). A quantitative Ty1 mobility assay was performed in the 1 Ty1 yeast strain containing empty vector (Vector), WT or H1Δ plasmids. The single element in the 1 Ty1 strain is marked with the retrotransposition indicator gene *his3-AI* [33]. A Ty1*HIS3* genomic insertion that occurs following splicing of the *AI* (artificial intron) will complement the *HIS3* deletion mutation present in the strain. Therefore, the number of His^+^ colonies generally reflect the level of Ty1 mobility. As expected for cells undergoing Ty1 CNC, the level of Ty1*his3-AI* mobility decreased about 15-fold from plasmid-based expression of p22 [13,14]. However, H1Δ and WT displayed similar levels of Ty1 mobility, suggesting that deleting the H1 hairpin does not affect Ty1 CNC despite the modest decrease in p22 observed in the 0 Ty1 strain (Figure 9B). Perhaps removing only one of the Gag binding sites in domain I of Ty1i RNA is not enough to affect CNC because Gag produced in the 1 Ty1 strain stabilizes Ty1 RNA through binding to other sites.

### 3.8. AUG1 is Exposed in a 3D Structural Model of Domain I RNA

**Figure 10:**
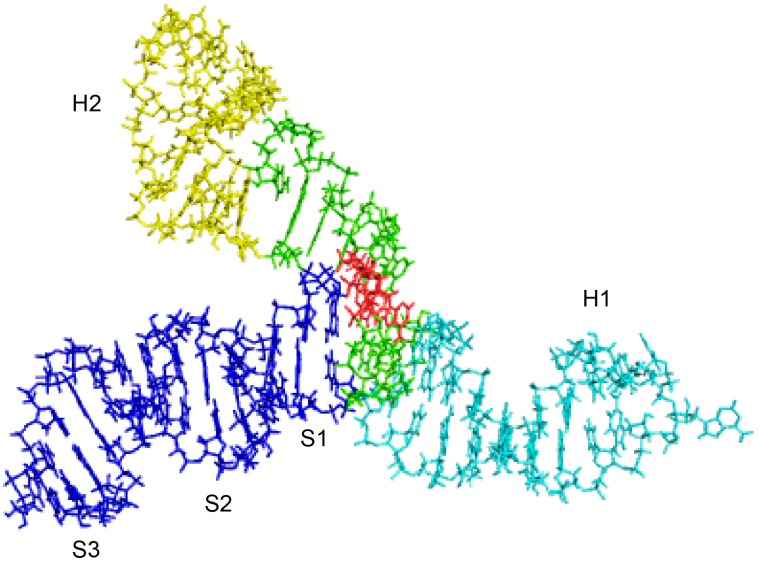
A 3D structure model of Ty1i RNA domain I. Structural elements are annotated: hairpin H1 (cyan), hairpin H2 (yellow), stem S1–3 (blue) and 3-way junction (green). AUG1 sequence is marked in red.

Our Ty1i RNA structural and functional studies indicate that the 3D structure of domain I is important for efficient p22 translation. However, determining the 3D structure of RNA in solution is challenging. Therefore, we combined chemical probing experiments to map RNA secondary (Figure 3) and tertiary structures using RNAComposer [34]. To reveal the tertiary fold of domain I of AUG1AUG2 RNA and support RNAComposer predictions [36], we also used hydroxyl radicals to produce strand breaks. This approach allows one to map solvent exposed regions of the nucleic acid backbone. This analysis predicted >100 different 3D structures of domain I and clustered them based on their agreement with the hydroxyl radical cleavage data and the energy of the final RNA 3D structure. The structures that best-fit the hydroxyl radical cleavage data allowed us to explain the gain in SHAPE reactivity of H2 apical loop nucleotides upon S1 stem destabilization in the AUG1GCG2 and AUG1GUG2 RNA mutants. Our models suggest that the H2 hairpin stem bends due to the presence of an internal loop containing unpaired C1051 and A1063, which causes an apical loop of H2 to be positioned close to the 3-way junction. Thus, disruption of junction geometry due to S1 unwinding is likely to affect H2 apical loop reactivity. The best models shared the common feature of coaxial positioning of the S1 stem and H1 hairpin. Such an organization of the 3-way junction places AUG1 on the surface of the molecule between hairpins H1 and H2, and may contribute to AUG1’s preferential use for initiating the translation of p22 (Figure 10). 

## 4. Conclusions

Translation initiation is the rate-limiting step of protein synthesis and is highly regulated by RNA binding factors and structural properties of the messenger RNA. This coordinated action allows cells to rapidly adapt to their environment without the need of de novo mRNA synthesis and transport from the nucleus to the cytoplasm [50]. In addition, a wide variety of viruses exploit variations in translation initiation to expand their coding capacity from a limited set of transcripts, including the use of alternative initiation codons and internal ribosome entry sites [17]. In the present work, we address how the Ty1 restriction factor p22 is translated from Ty1i RNA using a combination of structural and functional approaches. We show that two p22 initiation codons on Ty1i RNA are embedded in structural domain I, which is formed by an interaction between the 5′ UTR and the coding sequence. Our in vitro translation experiments show that both p22 initiation codons can be utilized but that AUG1 is used preferentially. We demonstrate that the structural integrity of Ty1i RNA is critical for the efficient expression of p22 from AUG1. Even small changes in the domain I sequence that disrupt its secondary and tertiary structure result in strong inhibition of p22 synthesis. Our studies have mapped two high affinity Ty1 Gag binding sites located in domain I of Ty1i RNA. Deletion of one of the binding sites leads to a decrease in the p22 level in vivo by destabilizing Ty1i RNA. Our work supports the hypothesis that structural motifs of domain I are not only important for the efficient translation of p22 protein but may also contribute to the stability of Ty1i RNA via interactions with Gag. Such interactions raise the possibility of an autogenous control loop where Gag positively controls the synthesis of p22, which in turn inhibits Gag function and mediates Ty1 CNC. However, more work will be required to understand how Gag binding to Ty1i RNA contributes to its stability. 

## Supplementary Materials

The following are available online at www.mdpi.com/1999-4915/9/4/74/s1, Figure S1: SHAPE reactivity AUG1GUG2 RNA mutant, Table S1: Primers used for construction of templates for in vitro transcription and reverse transcription, Table S2: Quantitation of the translation products from the gel in Figure 2, Data set S1: SHAPE data of AUG1AUG2 RNA.

## References

[1] Kim J.M., Vanguri S., Boeke J.D., Gabriel A., Voytas D.F. (1998). Transposable elements and genome organization: A comprehensive survey of retrotransposons revealed by the complete *saccharomyces cerevisiae* genome sequence. Genome Res..

[2] Curcio M.J., Lutz S., Lesage P. (2015). The Ty1 ltr-retrotransposon of budding yeast. Microbiol. Spectr..

[3] Feng Y.X., Moore S.P., Garfinkel D.J., Rein A. (2000). The genomic RNA in Ty1 virus-like particles is dimeric. J. Virol..

[4] Belcourt M.F., Farabaugh P.J. (1990). Ribosomal frameshifting in the yeast retrotransposon Ty: tRNAs induce slippage on a 7 nucleotide minimal site. Cell.

[5] Roth J.F., Kingsman S.M., Kingsman A.J., Martin-Rendon E. (2000). Possible regulatory function of the *saccharomyces cerevisiae* Ty1 retrotransposon core protein. Yeast.

[6] Mellor J., Fulton A.M., Dobson M.J., Roberts N.A., Wilson W., Kingsman A.J., Kingsman S.M. (1985). The Ty transposon of *saccharomyces cerevisiae* determines the synthesis of at least three proteins. Nucleic Acids Res..

[7] Pachulska-Wieczorek K., Le Grice S.F., Purzycka K.J. (2016). Determinants of genomic RNA encapsidation in the *saccharomyces cerevisiae* long terminal repeat retrotransposons Ty1 and Ty3. Viruses.

[8] Bourc'his D., Bestor T.H. (2004). Meiotic catastrophe and retrotransposon reactivation in male germ cells lacking Dnmt3L. Nature.

[9] Yoder J.A., Walsh C.P., Bestor T.H. (1997). Cytosine methylation and the ecology of intragenomic parasites. Trends Genet..

[10] Harris R.S., Dudley J.P. (2015). Apobecs and virus restriction. Virology.

[11] Drinnenberg I.A., Fink G.R., Bartel D.P. (2011). Compatibility with killer explains the rise of RNAi-deficient fungi. Science.

[12] Drinnenberg I.A., Weinberg D.E., Xie K.T., Mower J.P., Wolfe K.H., Fink G.R., Bartel D.P. (2009). RNAi in budding yeast. Science.

[13] Garfinkel D.J., Nyswaner K., Wang J., Cho J.Y. (2003). Post-transcriptional cosuppression of Ty1 retrotransposition. Genetics.

[14] Nishida Y., Pachulska-Wieczorek K., Blaszczyk L., Saha A., Gumna J., Garfinkel D.J., Purzycka K.J. (2015). Ty1 retrovirus-like element gag contains overlapping restriction factor and nucleic acid chaperone functions. Nucleic Acids Res..

[15] Saha A., Mitchell J.A., Nishida Y., Hildreth J.E., Ariberre J.A., Gilbert W.A., Garfinkel D.J. (2015). A trans-dominant form of gag restricts Ty1 retrotransposition and mediates copy number control. J. Virol..

[16] Tucker J.M., Larango M.E., Wachsmuth L.P., Kannan N., Garfinkel D.J. (2015). The Ty1 retrotransposon restriction factor p22 targets gag. PLoS Genet..

[17] Firth A.E., Brierley I. (2012). Non-canonical translation in RNA viruses. J Gen. Virol..

[18] Bolinger C., Boris-Lawrie K. (2009). Mechanisms employed by retroviruses to exploit host factors for translational control of a complicated proteome. Retrovirology.

[19] Pfingsten J.S., Kieft J.S. (2008). RNA structure-based ribosome recruitment: Lessons from the dicistroviridae intergenic region ireses. RNA.

[20] Kozak M. (1989). Circumstances and mechanisms of inhibition of translation by secondary structure in eucaryotic mRNArs. Mol. Cell. Biol..

[21] Sagliocco F.A., Vega Laso M.R., Zhu D., Tuite M.F., McCarthy J.E., Brown A.J. (1993). The influence of 5′-secondary structures upon ribosome binding to mRNA during translation in yeast. J Biol. Chem..

[22] Vega Laso M.R., Zhu D., Sagliocco F., Brown A.J., Tuite M.F., McCarthy J.E. (1993). Inhibition of translational initiation in the yeast *saccharomyces cerevisiae* as a function of the stability and position of hairpin structures in the mRNA leader. J. Biol. Chem..

[23] Babendure J.R., Babendure J.L., Ding J.H., Tsien R.Y. (2006). Control of mammalian translation by mRNA structure near caps. RNA.

[24] Kozak M. (1989). Context effects and inefficient initiation at non-AUG codons in eucaryotic cell-free translation systems. Mol. Cell. Biol..

[25] Kozak M. (1990). Downstream secondary structure facilitates recognition of initiator codons by eukaryotic ribosomes. Proc. Natl. Acad. Sci. USA.

[26] Kochetov A.V., Palyanov A., Titov, Grigorovich D., Sarai A., Kolchanov N.A (2007). AUG_hairpin: Prediction of a downstream secondary structure influencing the recognition of a translation start site. BMC Bioinform..

[27] Kozak M. (1986). Influences of mRNA secondary structure on initiation by eukaryotic ribosomes. Proc. Natl. Acad. Sci. USA.

[28] Blaszczyk L., Ciesiolka J. (2011). Secondary structure and the role in translation initiation of the 5′-terminal region of p53 mRNA. Biochemistry.

[29] Gorska A., Blaszczyk L., Dutkiewicz M., Ciesiolka J. (2013). Length variants of the 5′ untranslated region of p53 mRNA and their impact on the efficiency of translation initiation of p53 and its n-truncated isoform deltanp53. RNA Biol..

[30] Purzycka K.J., Legiewicz M., Matsuda E., Eizentstat L.D., Lusvarghi S., Saha A., Le Grice S.F., Garfinkel D.J. (2013). Exploring Ty1 retrotransposon RNA structure within virus-like particles. Nucleic Acids Res..

[31] Pachulska-Wieczorek K., Blaszczyk L., Biesiada M., Adamiak R.W., Purzycka K.J. (2016). The matrix domain contributes to the nucleic acid chaperone activity of HIV-2 Gag. Retrovirology.

[32] Purzycka K.J., Pachulska-Wieczorek K., Adamiak R.W. (2011). The in vitro loose dimer structure and rearrangements of the HIV-2 leader RNA. Nucleic Acids Res..

[33] Curcio M.J., Garfinkel D.J. (1991). Single-step selection for Ty1 element retrotransposition. Proc. Natl. Acad. Sci. USA.

[34] Popenda M., Szachniuk M., Antczak M., Purzycka K.J., Lukasiak P., Bartol N., Blazewicz J., Adamiak R.W. (2012). Automated 3D structure composition for large RNAs. Nucleic Acids Res..

[35] RNAComposer Automated RNA Structure 3D Modeling Server. http://rnacomposer.ibch.poznan.pl/.

[36] Biesiada M., Purzycka K.J., Szachniuk M., Blazewicz J., Adamiak R.W. (2016). Automated RNA 3D structure prediction with RNAcomposer. Methods Mol. Biol..

[37] Wilkinson K.A., Merino E.J., Weeks K.M. (2006). Selective 2′-hydroxyl acylation analyzed by primer extension (shape): Quantitative RNA structure analysis at single nucleotide resolution. Nat. Protoc..

[38] Deigan K.E., Li T.W., Mathews D.H., Weeks K.M. (2009). Accurate shape-directed RNA structure determination. Proc. Natl. Acad. Sci. USA.

[39] Reuter J.S., Mathews D.H. (2010). RNAstructure: Software for RNA secondary structure prediction and analysis. BMC Bioinform..

[40] Tijerina P., Mohr S., Russell R. (2007). DMS footprinting of structured RNAs and RNA-protein complexes. Nat. Protoc..

[41] Pachulska-Wieczorek K., Purzycka K.J., Adamiak R.W. (2006). New, extended hairpin form of the TAR-2 RNA domain points to the structural polymorphism at the 5′ end of the HIV-2 leader RNA. Nucleic Acids Res..

[42] Woodson S.A., Koculi E. (2009). Analysis of RNA folding by native polyacrylamide gel electrophoresis. Methods Enzymol..

[43] Araujo P.R., Yoon K., Ko D., Smith A.D., Qiao M., Suresh U., Burns S.C., Penalva L.O. (2012). Before it gets started: Regulating translation at the 5′ UTR. Comp. Funct. Genom..

[44] Mathews D.H. (2014). RNA secondary structure analysis using RNAstructure. Curr. Protoc. Bioinform..

[45] Huang Q., Purzycka K.J., Lusvarghi S., Li D., Legrice S.F., Boeke J.D. (2013). Retrotransposon Ty1 RNA contains a 5′-terminal long-range pseudoknot required for efficient reverse transcription. RNA.

[46] Dikstein R. (2012). Transcription and translation in a package deal: The tisu paradigm. Gene.

[47] Kozak M. (1991). A short leader sequence impairs the fidelity of initiation by eukaryotic ribosomes. Gene Expr..

[48] Kozak M. (2002). Pushing the limits of the scanning mechanism for initiation of translation. Gene.

[49] Garfinkel D.J., Tucker J.M., Saha A., Nishida Y., Pachulska-Wieczorek K., Błaszczyk L., Purzycka K.J. (2015). A self-encoded capsid derivative restricts Ty1 retrotransposition in Saccharomyces. Curr. Genet..

[50] Sonenberg N., Hinnebusch A.G. (2009). Regulation of translation initiation in eukaryotes: Mechanisms and biological targets. Cell.

