# Supplementary material for: Structure of Ty1 Internally Initiated RNA Influences Restriction Factor Expression
Source: Viruses. 2017 Apr 10;9(4):74. doi: 10.3390/v9040074 (PMC5408680; doi:10.3390/v9040074)
Supplement: Supplementary file 1 [file viruses-09-00074-s001.zip › supplementary revised/Table S1.pdf]

Primer name	Sequence 5'-3'
F-AUG1AUG2	**TAATACGACTCACTATA**GGGTCAAAGACATCCTATCC
F-AUG1GCG2	**TAATACGACTCACTATA**GGGTCAAAGACATCCTATCCGTTGATTATACGGATATCATGAAAATTCTTTCCAAAAGTATTGAAAAAGCGCAATCTGATACCCAAGAGGCAAACGACA
F-AUG1GUG2	**TAATACGACTCACTATA**GGGTCAAAGACATCCTATCCGTTGATTATACGGATATCATGAAAATTCTTTCCAAAAGTATTGAAAAAGUGCAATCTGATACCCAAGAGGCAAACGACA
F-AUG1AUG2(Δ5'UTR)	**TAATACGACTCACTATA**GGATATCATGAAAATTCTTTCC
F-AUG1AUG2(RND)	**TAATACGACTCACTATA**GGATCACTTCAAGACGACACACAATTCATCAGGATATCATGAAAATTCTTTCCAAAAG
F-AUG1AUG2(ΔH1)-A	GGATATCATGAAAATTCTTTCC
F-AUG1AUG2(ΔH1)-B	**TAATACGACTCACTATA**GGGTCAAAGACATCCGGATATCATGAAAATTCTTTCC
F-AUG1AUG2*-A	CCTATCCGTTGATTATACGGATATCATGAAAATTCTTTCCAAAAGTATTGAAAAAGCGCAATCTGATACCCAAGAGGCAAAAAAAATGCAAACCCTGGCAAATTTGCAATATAATGG
F-AUG1AUG2*-B	**TAATACGACTCACTATA**GGGTCAAAGACATCCTATCCGTTGATTATACGGATATC
F-AUG1^{stop}AUG2	**TAATACGACTCACTATA**GGGTCAAAGACATCCTATCCGTTGATTATACGGATATCATGAAAATTCTTTCCAAAAGTATTTGAAAAAATGCAATCTGATACCCAAGAGGCAAACGACA
F-AUG1^{frs}AUG2	CTATCCGTTGATTATACGGATATCATGAAAATTCTTTATCCAAAAGTATTGAAAAAATGCAATCTGATACCCAAGAGGCAAACGACATTGAGACCCTGGCAAATTTGCAATATAATGG
F-241-Gag	**TAATACGACTCACTATA**GGAGGAGAACTTCTAGTATATTC
F-816-Gag	**TAATACGACTCACTATA**GGTTAACCTCACCTAATGAC
F-953-Gag	**TAATACGACTCACTATA**GGTATAACACTTTTCAAATATTTGCTCCC
F-241-Gag(Δ5'UTR)	**TAATACGACTCACTATA**GTCAACAATGGAATCCCAACAATTATCTCAAC
R-AUG1AUG2	TTTACTGTAGATTCAGTAAGTTTCTGG
