# Supplementary material for: Structure of Ty1 Internally Initiated RNA Influences Restriction Factor Expression
Source: Viruses. 2017 Apr 10;9(4):74. doi: 10.3390/v9040074 (PMC5408680; doi:10.3390/v9040074)
Supplement: Supplementary file 1 [file viruses-09-00074-s001.zip › supplementary revised/Table S2.pdf]

Table S2. Quantitation of the translation products from the gel in Figure 2. The band intensities were normalized to the level of translation from the AUG1AUG2 RNA.

	AUG1	AUG2
RNA AUG1AUG2	100%	
RNA AUG1AUG2*	22%	4%
RNA AUG1^{stop}AUG2	-	9%
RNA AUG1^{frs}AUG2	51%	8%
