# Supplementary figures and images for: Structure of Ty1 Internally Initiated RNA Influences Restriction Factor Expression
Source: Viruses. 2017 Apr 10;9(4):74. doi: 10.3390/v9040074 (PMC5408680; doi:10.3390/v9040074)

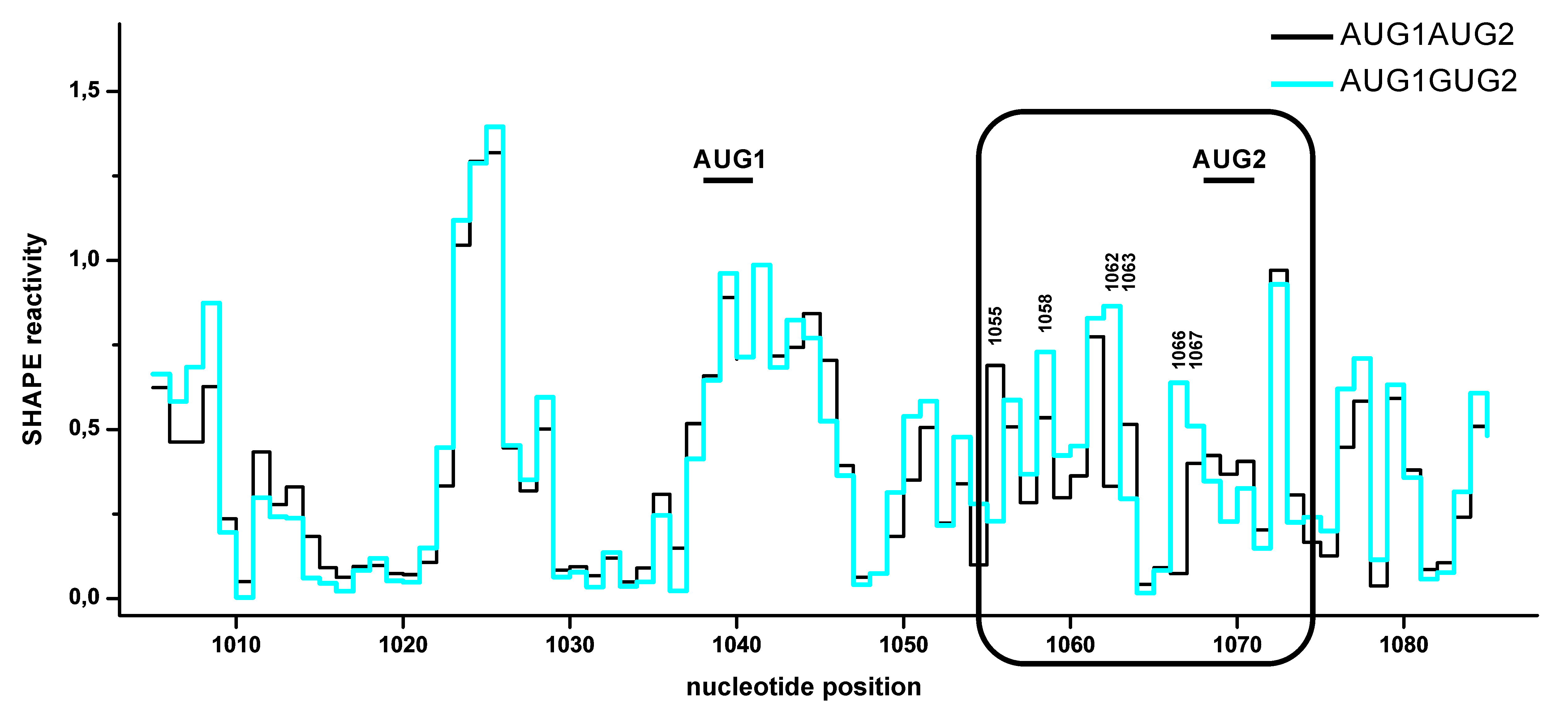

Supplement: Supplementary file 1 [file viruses-09-00074-s001.zip › supplementary revised/Figure S1 v2.tif]
